# Developing Person-Centred Dental Care: The Perspectives of People Living in Poverty

**DOI:** 10.3390/dj8030082

**Published:** 2020-08-03

**Authors:** Nioushah Noushi, Christophe Bedos

**Affiliations:** 1 Faculty of Dentistry, McGill University, Montréal QC H3A 1G1, Canada; christophe.bedos1@mcgill.ca; 2 School of Public Health, Université de Montréal, Montréal QC H3N 1X9, Canada

**Keywords:** person-centred dentistry, patient-centred care, biopsychosocial health, whole person care, qualitative methods, dental care

## Abstract

Dentistry has seen a slow trend toward person-centred care (PCC), with most approaches developed by scholars who have tried to progress away from disease-centred care. Unfortunately, the perspectives and experiences of underprivileged people have not been considered in the development of these approaches. Our objective was thus to understand underprivileged people’s experiences and expectations about dental care and contribute to the development of person-centred dentistry. We conducted a qualitative descriptive study with a sample of 13 people living in poverty. We used a maximum variation sampling strategy and selected them among the users of a free dental clinic in Montreal, Canada. We conducted semi-structured interviews that we audio recorded, transcribed verbatim, and thematically analysed. Our main finding is that participants wanted to feel human and respected by dentists. More specifically, they wanted to be more involved in the dental care process through quality time and empathetic conversations with the dentist. They also wished for an exchange of information free of technical terms and built on mutual trust. In conclusion, person-centred dental care models should emphasize empathy, trust, and quality care beyond technical skills. Clinicians should provide comprehensive information in dental encounters and treat their patients as whole persons.

## 1. Introduction

The past decade has seen significant strides away from the paternalistic model within dentistry [1,2,3]. Different governing bodies have encouraged patient-centred care and a few frameworks have been developed in Canada and the UK [4,5,6,7,8,9]. These person-centred care (PCC) models emphasize communication during clinical encounters and the importance for dental professionals to understand patients’ perspectives, their expectations, and their needs. They also stipulate that dentists and their patients should share power in order to choose the best treatment options.

In Canada, for instance, Apelian et al. (2014) proposed a model based on humanistic values and humility [4]. According to the authors, dentists should understand their patients’ illness and disease, elaborate treatment plans in partnership with the latter, and conduct interventions that are mindful of patients’ fears, pace, and expectations [4]. In the UK, Scambler and Asimakopoulou describe patient-centred care as a process based on the provision of information and choice preceding four foundational components: exploring disease and its context, considering the patient as a whole person, feeling empathy and compassion, and finding common ground and sharing responsibility [8,10,11].

It needs to be noted that these models have been developed by clinicians and researchers. Despite the patients being at the centre of these concepts, their voices have not been sufficiently considered and addressed yet. We therefore lack information on people’s preferences about dental care and how these fit into the conceptualization of person-centred dental care. In sum, people, especially those who face challenges to access dental services, should be invited to express their needs and expectations about dental encounters and contribute to the development of person-centred care in dentistry.

Our objective was thus to understand the experiences and expectations of underprivileged people about dental care and contribute to the development of person-centred care in dentistry.

## 2. Methodology 

We conducted a qualitative descriptive study as defined by Sandelowski [12,13]. This methodology was conducive to thorough descriptions of the perspectives of adults regarding dental care [12]. The goal of qualitative description is to produce a contextualized and comprehensive summary of events as they were experienced, described, and defined by study participants [12]. 

Our population of interest was people who are often excluded from the privatized Canadian dental care system in Montreal, Canada due to their socioeconomic status. These participants frequented free dental clinics since they could not afford dental care and did not benefit from a dental insurance or social assistance. More specifically, we recruited participants who attended the free Jim Lund Dental Clinic (JLDC) in Montreal. The JLDC is a clinic offering free dental care to low income and homeless Montrealers without access to dental insurance, which often encompasses a large immigrant population reflective of the Montreal community [12,14]. We used a purposive sampling strategy during recruitment and applied a maximum variation technique to explore the perspectives of various adults regarding dental care [12]. To be included in the study, people had to be able to speak French or English, be at least 18 years old, and consult the Jim Lund Dental Clinic. For the purposes of this study, we considered the definitions and conceptualizations of PCC from the researchers stated above but chose an approach that allowed our participants to define the concept as they saw fit through their experiences.

This study was conducted per the ethical principles stated in the declaration of Helsinki (2013). We obtained ethics approval from the McGill Institutional Review Board (IRB) at the Faculty of Medicine, McGill University on 20 July 2016, IRB Study Number A06-B27-16A. Prior to being interviewed, participants were asked to sign a consent form explaining the details of the study. The form emphasized that their participation was voluntary and that they could opt out of the study at any time without any consequences. 

We conducted semi-structured individual interviews at various locations as per the preferences of the participants, including rooms in the McGill University Faculty of Dentistry building, rooms in the Jim Lund Dental Clinic, or a café/public eatery. During the interviews, which were conducted in French or English according to participants’ choice, we invited them to describe their expectations and experiences of dental care, how they felt about them, and what meaning they gave to these experiences. Our interview guide included open-ended and probing questions pertaining to each participants’ personal history, experiences with the dental care process, and experiences with public and private dental clinics. Additionally, we discussed their interactions with clinicians and the clinic, their perspectives about person-centred care, as well as their time at the Jim Lund Dental Clinic. This study aimed to recruit 10 to 15 participants and reached data saturation after the 10th interview—the point after which the information we gathered was redundant with previous data and no new information emerged. We thus decided to stop recruiting participants after the 13th interview. All interviews were audio-recorded and transcribed verbatim.

We conducted a thematic analysis of the data following the six-step approach described by Braun and Clarke [15]. Thematic analysis is an exploratory approach [16] that helps identify, analyse and report patterns, otherwise known as themes, within the data collected during the study [15]. We used MAXQDA Standard 12 (12.2.1; VERBI GmbH 2016, Berlin, Germany) to organize the inductively emerging codes of all transcripts. We grouped the codes and sub-codes together based on their similarities and differences by using memos kept throughout the coding process. We then identified emerging themes and used MAXQDA to extract relevant quotes from the transcripts to illustrate our findings.

## 3. Findings

The participants ranged from 29 to 72 years of age (Table 1). Four were of Canadian origin, while the other nine originated from seven countries: Russia, India, Syria, Morocco, Moldova, Cameroon and Peru. The majority of participants were either unemployed or retired. All faced economic challenges, which was a central criterion to receive free treatment at the Jim Lund Dental Clinic. 

“Wanting to Feel Human and Respected” was a major theme that emerged from the analyses. It included four interrelated dimensions that we will describe in the next paragraphs: Wanting to Feel Cared for by the Clinician and the Clinic, Wanting to Receive Quality Service, Wanting to Build Trust with Clinicians and the Clinic, and Wanting Sufficient and Appropriate Information. It is worth noting that these dimensions occasionally overlap based on the complexities of human experiences that cannot always be strictly categorized into a single theme or dimension.

### 3.1. Wanting to Feel Cared for by the Clinician and the Clinic

By being cared for, participants meant they wanted a respectful clinical environment that would treat them as whole people and would not focus strictly on their dental ailments. For them, this feeling of being cared for depended on qualities that clinicians and their staff should have, such as kindness and the ability to provide them with adequate information about their oral health.

You want friendly people too, like the receptionist and you know, you need to feel that you are being cared for, you are, you are a person and you are somebody.

*[Interview #2]*

While sharing positive and negative experiences in dental clinics, participants explained how dentists’ lack of certain qualities, such as friendliness and respect, could lead to unproductive and potentially harmful clinical visits. A participant, for instance, criticized shaming tactics that would reveal clinicians’ lack of empathy. He argued that they should be avoided because they create an unsafe space where the patient does not feel cared for.

He wasn’t rude or anything like that, it was just the way he spoke to you, you know? You would, you would know very well when he was disappointed. it was more, very much like a father figure, like he cared, he just, all the work he did when I was a kid and he would see it go away. So, he was, he was pretty stern and open about his thoughts, but he wasn’t, he wasn’t bad in any ways, just very straightforward. […] After a while, I was just too ashamed to look down the guy who’s been working on my teeth my whole life and for him to see what happened. I was just, I couldn’t, couldn’t get myself to do it. […] I guess maybe it’s like an old school versus new school. Because I know they have bedside manners so maybe he comes from a different, a different time when it wasn’t such a thing, but no, it was very much more like business than interpersonal, for sure.

*[Interview #4]*

Some participants also denounced Western society’s culture of “busyness” that also applied to dentistry. According to them, it leads patients to feel neglected, forgotten, and undervalued. Participants illustrated this phenomenon by explaining that certain clinicians overbook and rush through dental appointments, thereby making them feel like a burden.

Today it’s fast, fast, fast, fast, fast, fast, you know? That’s the problem, it’s fast, fast, fast, so we forget, we forget the person sometimes. The person is important.

*[Interview #7]*

[...] the other dentists, the focus they have, what they have is that, it just on a surface level, just like they want to do, make it quick as much as possible and then let you go, you know? And you also feel kind of not, because you see that, you know, you are taking their time, when you feel that, if you have, I have questions or the thing you want just, you know, take time.

*[Interview #3]*

### 3.2. Wanting to Receive Quality Service

Participants’ comments suggested that the dental profession should hear people’s desire for quality time with their clinician. These interactions, according to them, should be based on dentists’ empathy and sensibility to each individual’s needs.

Participants acknowledged that, since their ability to access dental care in a privatized system was limited, their experiences mostly related to recent visits at the free Jim Lund Dental Clinic. They stated that the quality of care inevitably included dentists’ technical skills. This said, they also emphasized how quality care encompasses clinicians’ personal characteristics, such as empathy and sensitivity to patients’ needs. One participant described how the clinician treated him with empathy and was considerate when providing care:

He came, he touched me in a way that even if it’s not his body, and he doesn’t feel anything, he knows that I feel things and he made sure that I wouldn’t feel anything, that I wouldn’t be hurt, ok.

*[Interview #12]*

A participant remarked on the potential tensions that exist between the quality of services and the business side of dentistry. He explained that, in his experience and interpretation, private clinics provided a basic and superficial level of care and often disregarded other personal issues since they focused on profit. In such a context, clinicians may forgo the quality of the interaction with the patient in order to maximise profitability.

So, the other places, this is the problem I see that when there’s money-oriented, then the quality may not be, you know, matter. So that’s why they are doing kind of a surface job and then also, they don’t go in seeing the, or they don’t take time in seeing other, you know, issues.

*[Interview #3]*

### 3.3. Wanting to Build Trust with Clinicians and the Clinic

All the participants agreed that trusting the dental staff was integral to having positive dental experiences. Some described the conditions of building trust as the clinicians being interested in them as people, being mindful of their specific needs, and taking the time to ask questions about more than just dental health.

Check, well when you come back, just, when you come back from one appointment and it’s weeks later, just when they ask you how you’ve been feeling, or just recapping on the mlast appointment and the time in between, I think just kinda prodding the questions, getting people to express it, that, I think that helps with it too. Yeah. Just that social interaction in general builds trust, for sure. Even if it’s just checking up like “Oh, hey, you know, did the stitches come out ok?” It’s just putting in my head that “Oh, he remembers exactly what he did four weeks ago, I’m not just another person laying on the bed”, I guess. Yeah, that’s, that’s how I look at it, and I put it in the same boat as trust.

*[Interview #4]*

One participant described an experience that highlighted the sensitivity of a previous dentist and how it resulted in a relationship of mutual trust. She explained that this dentist would send a taxi to transport her to his private clinic when he realized that she was unable to pay for her transportation or pay upfront for her treatments. This situation showed the clinician’s empathy to her circumstances, his confidence in her, and his capacity to take active steps to improve her experience. The willingness of the clinician to take this step created the foundation for a trusting patient-dentist relationship.

Trust, build trust, it took a long time, the fact that they were very patient with me, there was a time when I didn’t have money to pay but like I told you, not only did they send a taxi for me, but they would say ‘Ok, you need a crown, you don’t have money, we trust you, you can pay 100$ a month’, I don’t know how else we build trust.’”

*[Interview #9]*

Our analyses suggest that by providing care that focuses on each individual and their specific needs, dentists build trust during their clinical encounters. For instance, a participant described how providing services with kindness and benevolence—as opposed to financial motives—could support a trusting relationship between the patient and the clinician.

When you see that they want to help you, right? Without, with no other motive, just help you, there’s no other motivation. That really builds your trust.

*[Interview #3]*

### 3.4. Wanting Sufficient and Appropriate Information

Communication was a nearly ubiquitous topic of discussion throughout this study. It was indeed essential for the participants to receive information from their clinician in an environment that supported an enriching exchange. This implied that dentists should not rush the clinical encounter and should ensure that the patient has adequate opportunity to ask questions in a respectful context. Participants explained that they would feel more comfortable in their interaction with the clinician if sufficient time was taken to establish clear lines of communication. For example, a participant described how talking about topics outside of dentistry with his dentist comforted him during a potentially stressful clinical experience.

I think it’s just communicating with the person gives people comfort in general, […] maybe I’m thinking about it more like a bedside manner, it’s just something you do as a, as an extra to kind of put people at ease.

*[Interview #4]*

Participants emphasized the need to get appropriate information about their dental care in layperson’s terms since technical terminology and jargon were not sufficiently informative and left room for potential misunderstandings. Therefore, participants recommended professionals to be aware of the way they provide information to ensure that they are well understood.

By somebody sticking something in my mouth and going ‘10 inches, eight, three, two, one [said in a robotic voice]’, and talking to a dental hygienist means nothing to me. By him saying to me ‘You have periodontal’, means nothing to me.

*[Interview # 9]*

Participants also mentioned how they wanted the clinician to explain the various care options they have based on their needs and in terms they can understand. This level of communication would be paired with the possibility to deliberate with their dentist, discuss their preferences, and then jointly choose the best option according to their needs. Providing care in this way supports each person’s role in the patient-dentist relationship through shared decision-making.

[...] together is better. To sit down and then say, because first you always have ‘How much will it cost?’, you will decide what you will do, ok, you will decide the price as well [laughter]. That is one thing, you know, but to come ‘Look, you have this, this, and this possibility’, let’s say. ‘You have the choice between repairing the tooth with paste and then it’s dried and it becomes hard, paff, it’s done. I can do you a nice job with that, or we extract the tooth and we do [...] an implant’.

*[Interview #12]*

## 4. Discussion

This study improves our understanding of underprivileged people’s experiences and expectations of dental care and contributes to the development of person-centred care in dentistry. Additionally, our findings add the perspectives of people living with poverty to existing models and frameworks of PCC [1,2,3,8,10]. More specifically, our findings reinforce the pertinence of some models, especially those proposed by Mills et al. and Scambler et al., which emphasize mutual trust, holistic care, empathy, and provision of information. Our study also supports the relevance of the three principles brought forth by Apelian et al. leading to a “humanist equal-powered patient-dentist encounter” [1,2,3,8,10]. Participants wanted to feel cared for by clinicians, be treated as human beings, and not reduced to their dental ailments. They expected relationships based on mutual respect and trust and wished to be able to discuss with the dentist, be understood, and become actors in the decision-making process. Our study echoes the work of Raja et al., who reported the need to feel cared for by people feeling dehumanized when not “seen as an entire human being in oral health settings” [18]. It is also in tune with recent initiatives to address the needs of underprivileged people, such as inclusive dentistry [19] and social dentistry [7,20,21]. The desire to be treated in a humanized way is not singular to dental encounters; it reflects people’s experiences in a broader social context that perpetuates the oppression of people living in poverty. People seek to escape a “poverty stigma” [22] and instances of “othering” and look for caring, respectful, and holistic life experiences [19].”

It is important to note that this study reflects the experiences and perspectives of a small and specific population in the Montreal community. Considering the nature of qualitative research, readers should be careful in applying our findings in their own context. This being said, we consider that our study had an appropriate sample size and provided rich data that could not have been produced with quantitative approaches [23]. 

Our study demonstrates that people want more than just dental or surgical procedures from dentists. Thus, conversations should not be limited to biomedical information but also include casual discussions that support trust and better rapport with patients. Mills et al. highlighted the relationship between rapport and trust by finding that some patients used “engagement, rapport and shared values or beliefs” as a way of choosing a dentist [3,24,25]. Raja et al. also underlined the importance of empathy and rapport and their role in the patient-dentist relationship [18]. Our study further shows that communication should be jargon-free. As other authors also mentioned [3,18], people want information they can understand in order to make informed decisions in partnership with their clinicians. 

Our findings also highlight that people may associate quality care to clinicians’ traits, especially empathy, which is a concept that is central to person-centred health and dental care [16,24]. Mills et al. and Karydis et al. also reported that patients greatly value a dentist’s empathetic approach [25]; however, they did not always directly associate it with quality service [3,26]. Another emerging trait about quality dental care was clinicians’ sensitivity towards patients, a quality that study participants mostly observed in the free dental clinic they recently attended. Sensitivity is associated with empathy in the literature since it highlights the clinician’s ability to understand and be aware of patients’ feelings [27]. Study participants also linked empathy and sensitivity with trust, which they considered as an essential element for a positive dental care experience. 

**Figure 1:**
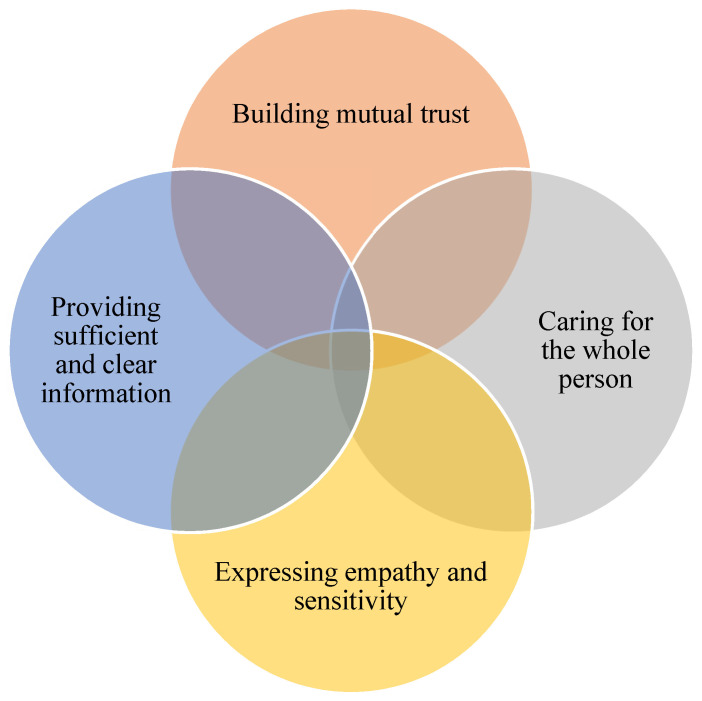
Important aspects to include in person-centred models to support respect and humanity during clinical encounters.

By uncovering the perspectives of people who often feel excluded from the dental care system, our study contributes to the development of person-centred care in dentistry. Figure 1 summarizes several elements that person-centred models could include and that dental professionals need to reflect upon: Building mutual trust; Caring for the whole person; Expressing empathy and sensitivity; and Providing clear and sufficient information. 

## 5. Conclusions

We invite dental educators and dental care professionals to take into account the elements we highlighted in this article when providing person-centred care. Our study underlines some important similarities in the disparate experiences of people living with poverty with regards to accessing dental care services in a privatized system: People seeking dental care want empathetic, respectful, and holistic care supported by clear and informative communication. We also encourage researchers to explore the perspectives of different populations, such as people with disabilities but also children and the elderly.

## Figures and Tables

**Table 1:** Sociodemographic characteristics of participants.

Dimensions (Characteristics)	Categories	Number of Participants
Gender	Female	7
	Male	6
Age (years)	18–29	1
	30–49	9
	50–69	2
	70–80	1
Marital status	Married	9
	Divorced/Widowed	3
	Single	1
Education level	High School/CEGEP *	6
	Undergraduate degree	7
Employment status	Employed	4
	Unemployed	7
	Retired	2

* CEGEP (Collège d’enseignement général et professionnel) is a pre-university college within the Quebec education system [17].

## References

[1] Mills I., Frost J., Moles D.R., Kay E. (2013). Patient-centred care in general dental practice: Sound sense or soundbite?. Br. Dent. J..

[2] Mills I., Frost J., Cooper C., Moles D.R., Kay E. (2014). Patient-centred care in general dental practice-a systematic review of the literature. BMC Oral Health.

[3] Mills I., Frost J., Kay E., Moles D.R. (2015). Person-centred care in dentistry-the patients’ perspective. Br. Dent. J..

[4] Apelian N., Vergnes J.-N., Bedos C. (2014). Humanizing clinical dentistry through a person-centred model. Int. J. Whole Pers. Care.

[5] Apelian N., Vergnes J.N., Hovey R., Bedos C. (2017). How can we provide person-centred dental care?. Br. Dent. J..

[6] Department of Health (2011). Dental Quality and Outcomes Framework.

[7] Bedos C., Apelian N., Vergnes J.-N. (2020). Towards a biopsychosocial approach in dentistry: The Montreal-Toulouse Model. Br. Dent. J..

[8] Scambler S., Asimakopoulou K. (2014). A model of patient-centred care–turning good care into patient-centred care. Br. Dent. J..

[9] Association of Canadian Faculties of Dentistry (2016). ACFD Educational Framework for the Development of Competency in Dental Programs.

[10] Scambler S., Gupta A., Asimakopoulou K. (2015). Patient-centred care–what is it and how is it practised in the dental surgery?. Health Expect..

[11] Asimakopoulou K., Gupta A., Scambler S. (2014). Patient-centred care: Barriers and opportunities in the dental surgery. Community Dent. Oral Epidemiol..

[12] Sandelowski M. (2000). Whatever happened to qualitative description?. Res. Nurs. Health.

[13] Sandelowski M. (2010). What’s in a name?. Qualitative description revisited. Res Nurs Health.

[14] Patton M.Q. (1990). Qualitative Evaluation and Research Methods.

[15] Braun V., Clarke V. (2006). Using thematic analysis in psychology. Qual. Res. Psychol..

[16] Dwamena F., Holmes-Rovner M., Gaulden C.M., Jorgenson S., Sadigh G., Sikorskii A., Lewin S., Smith R.C., Coffey J., Olomu A. (2012). Interventions for providers to promote a patient-centred approach in clinical consultations. Cochrane Database Syst. Rev..

[17] General and Vocational Colleges Act. http://legisquebec.gouv.qc.ca/en/ShowDoc/cs/C-29.

[18] Raja S., Shah R., Hamad J., Van Kanegan M., Kupershmidt A., Kruthoff M. (2015). Patients’ perceptions of dehumanization of patients in dental school settings: Implications for clinic management and curriculum planning. J. Dent. Educ..

[19] Freeman R., Doughty J., Macdonald M.E., Muirhead V. (2020). Inclusion oral health: Advancing a theoretical framework for policy, research and practice. Community Dent. Oral Epidemiol..

[20] Bedos C., Apelian N., Vergnes J.-N. (2018). Social dentistry: An old heritage for a new professional approach. Br. Dent. J..

[21] Bedos C., Apelian N., Vergnes J.N. (2018). Time to develop social dentistry. Jdr Clin. Transl. Res..

[22] Reutter L.I., Stewart M.J., Veenstra G., Love R., Raphael D., Makwarimba E. (2009). ‘Who do they think we are, anyway?’: Perceptions of and responses to poverty stigma. Qual. Health Res..

[23] Bedos C., Levine A., Brodeur J.-M. (2009). How people on social assistance perceive, experience, and improve oral health. J. Dent. Res..

[24] Mills I.J. (2017). A person-centred approach to holistic assessment. Prim. Dent. J..

[25] Mills I.J. (2018). Through the patient’s eyes-the importance of person-centred care in oral cancer. Br. Dent. J..

[26] Karydis A., Komboli-Kodovazeniti M., Hatzigeorgiou D., Panis V. (2001). Expectations and perceptions of Greek patients regarding the quality of dental health care. Int. J. Qual. Health Care.

[27] Wallace B.B., MacEntee M.I. (2012). Access to dental care for low-income adults: Perceptions of affordability, availability and acceptability. J. Community Health.

